# Flocculating performance of a bioflocculant produced by *Arthrobacter humicola* in sewage waste water treatment

**DOI:** 10.1186/s12896-017-0375-0

**Published:** 2017-06-12

**Authors:** Mayowa Oladele Agunbiade, Esta Van Heerden, Carolina H. Pohl, Anofi Tom Ashafa

**Affiliations:** 1 Phytomedicine and Phytopharmacology Research Group, Department of Plant Sciences, University of the Free State, Qwaqwa Campus, P. Bag X13, Phuthaditjabha, 9866 South Africa; 2 Department of Microbial, Biochemical & Food Biotechnology, University of the Free State, P.O. Box 339, Nelson Mandela Drive, Bloemfontein, 9301 South Africa

**Keywords:** Purified bioflocculant, *Arthrobacter humicola*, Sewage waste water, FTIR, Flocculating activity

## Abstract

**Background:**

The discharge of poorly treated effluents into the environment has far reaching, consequential impacts on human and aquatic life forms. Thus, we evaluated the flocculating efficiency of our test bioflocculant and we report for the first time the ability of the biopolymeric flocculant produced by *Arthrobacter humicola* in the treatment of sewage wastewater. This strain was isolated from sediment soil sample at Sterkfontein dam in the Eastern Free State province of South Africa.

**Results:**

Basic Local Alignment Search Tool (BLAST) analysis of the nucleotide sequence of the 16S rDNA revealed the bacteria to have 99% similarity to *Arthrobacter humicola strain R1* and the sequence was deposited in the Gene bank as *Arthrobacter humicola* with accession number KC816574.1. Flocculating activity was enhanced with the aid of divalent cations, pH 12, at a dosage concentration of 0.8 mg/mL. The purified bioflocculant was heat stable and could retain more than 78% of its flocculating activity after heating at 100 °C for 25 min. Fourier Transform Infrared Spectroscopy analysis demonstrated the presence of hydroxyl and carboxyl moieties as the functional groups. The thermogravimetric analysis was used to monitor the pyrolysis profile of the purified bioflocculant and elemental composition revealed C: O: Na: P: K with 13.90: 41.96: 26.79: 16.61: 0.74 weight percentage respectively. The purified bioflocculant was able to remove chemical oxygen demand, biological oxygen demand, suspended solids, nitrate and turbidity from sewage waste water at efficiencies of 65.7%, 63.5%, 55.7%, 71.4% and 81.3% respectively.

**Conclusions:**

The results of this study indicate the possibility of using the bioflocculant produced by *Arthrobacter humicola* as a potential alternative to synthesized chemical flocculants in sewage waste water treatment and other industrial waste water.

## Background

The discharge of untreated or partially treated waste water accounts for the majority of health related issues in human beings and aquatic life forms. When pollutants from waste water are discharged into natural water bodies, the water bodies become contaminated and toxic and may consequently pose a major threat to human beings. Water contains about 78% of the earth’s surface which is a source of life and energy. However millions of people doesn’t have access to safe water for drinking purposes and human utilization as a result of human negligence [1]. Hence, toxic and polluted material from the waste water must be well treated or removed prior to discharge into the environment [2]. Although, settling separation techniques has been widely adopted in most factories to recover suspended solid materials in waste water, the process is however time consuming and the separation efficiency is low. Consequently, the need for the use of flocculants which appear to be gaining global acceptance for various industrial processes such as treatment of potable water, waste water treatment and downstream processes in fermentation industries has been well appreciated [3]. A review of the literature shows that the use of inorganic and organic flocculants in various industrial and wastewater treatment processes is increasing [4–7]. However, despite the fact that organic and inorganic synthetic flocculants are cost effective, they are non-biodegradable and have been implicated in health related issues. For instance, polyacrylamide that contain acrylamide monomers are neurotoxic and carcinogenic [8]. Also, aluminium, which is a major component of polyaluminium chloride is known to be a contributing factor in Alzheimer’s syndrome. Based on the adverse health related issues attributed to the usage of chemically synthesized flocculants in the process of flocculation, there is need to employ microbial flocculants which are biodegradable and environmentally friendly that will serve as alternatives to chemical flocculants. Biodegradable bioflocculants have been isolated from different environments as natural byproducts of the growth of certain bacteria, fungi, algae and actinomycetes during their growth [9, 10]. Bioflocculants enhance the formation and settlement of sludge treatment systems and have the potential to provide better applications in relevant industries and wastewater treatment [11, 12]. Microbial flocculants with different functional properties, particularly those that are mainly polysaccharide, have been found to be useful as stabilizers, film forming agents, water retention agents, lubricants and friction reducers in many industries like textiles, adhesives, paper, paint, food, pharmaceuticals, cosmetics, detergents and in laundry products.

The genus *Arthrobacter*, found in the soil, is an obligate aerobic bacterium. This genus has found biotechnological importance in degrading organic pollutants such as phenols, chlorophenols, benzene, toluene, ethylbenzene, xylene and phenanthrene [13, 14]. It has also been reported as a potential microbial flocculant in flocculation. In spite of the flocculating potentials of this genus, there is dearth of information on the specific bioflocculant produced by *Arthrobacter humicola* for sewage waste water treatment. Hence, this study optimized the culture conditions and evaluated the flocculating potential of *Arthrobacter humicola* in sewage waste water treatment.

## Methods

### Collection of sample and isolation of bioflocculant-producing microorganism

Soil sediment samples were collected aseptically from Sterkfontein dam in the Eastern Free State Province of South Africa and transported to the laboratory in a cooler box containing an ice pack. Exactly 20 grams of each sediment sample was air-dried at room temperature for two to three days, crushed, sieved through a 2 cm mesh and kept refrigerated (4 °C) until use. The cultivation of actinomycetes from the processed sediment samples was done as previously described [15]. This involved the use of Yeast Malt Extract agar (YMA) (4 g/L yeast extract, 10 g/L malt extract, 4 g/L glucose, 16 g/L bacteriological agar) supplemented with 50 mg/L cyclohexamide and 20 mg/L nalidixic acid to inhibit the growth of bacteria and fungi species respectively. Subsequently, an aliquot (100 μL) of the sediment sample suspension was spread over the cultivation medium and incubated at 28 °C for two weeks. Typical colonies were purified by streaking on fresh YMA plates.

### Screening and culturing of bioflocculant-producing actinomycetes

The adapted protocol of Xia *et al.* [16] was used to screen the ten isolated actinomycete strains for bioflocculant production. The isolates were inoculated into 250 mL Erlenmeyer flask containing 50 mL of screening medium [20 g/L glucose, 0.5 g/L urea, 0.5 g/L yeast extract, 0.2 g/L (NH_4_)_2_SO_4_, 0.1 g/L NaCl, 0.2 g/L MgSO_4_.7H_2_O, 5 g/L K_2_HPO_4_, 2 g/L KH_2_PO_4_] and incubated (30 °C, 160 rpm) on a rotary shaker for 48 h. This pre-culture was then used as the standard inoculum for subsequent experiments. Erlenmeyer flasks (250 mL) containing 50 mL production medium (10 g/L glucose, 1 g/L yeast extract, 0.3 g/L MgSO_4_.7H_2_O, 5 g K_2_HPO_4_, 2 g/L KH_2_PO_4_ was inoculated with 100 μL of the pre-culture suspension and further incubated (25 °C, 130 rpm) in a rotary shaker for 48 h. After incubation, cell free culture supernatants were obtained by centrifugation and used to determine bioflocculant activity against kaolin clay suspension.

### Determination of flocculating activity

Flocculating activity was determined using the protocol of Kurane et al. [17]. A volume of 100 mL of kaolin clay suspension (4 g/L) was added to 2 mL of the supernatant and 3 mL of calcium chloride (1% w/v) in 250 mL conical flasks. The mixture was shaken thoroughly for 30 s and gently poured into 100 mL measuring cylinder and allowed to stand for 5 min. Following this, the optical density (OD) of the clarifying solution was measured using a spectrophotometer (UV/Visible Biowave II and Biowave II^+^ England) at 550 nm. The control experiment was conducted in the same way, but cell free supernatant was replaced with 2 mL of the production medium. The flocculating activity (FA) was calculated using the equation;

$$ F A\left(\%\right)=\frac{B- A}{B}\times 100 $$

Where: A and B are the respective absorbance of the sample and control experiment at 550 nm.

The strain that displayed the highest flocculating activity was selected for further investigation.

## Identification of organism

### DNA extraction

The genomic DNA of the strain was extracted using a ZR fungal/bacteria DNA preparation kit according to the manufacturer’s instructions.

### Amplification of 16S rRNA gene

The 16S rRNA gene was amplified using universal primers 27f: (5^1^ - GAGTTTGATCCTGGCTCAG - 3^1^) 1492r: (5^1^ – GGTTACCTTGTTACGACT - 3^1^) Lane [15]. PCR amplification was carried out in 20 μL reaction volume containing 10 μL of Econo PCR master mix, 1 mM of each primer, 1 μL of template DNA. Subsequently, 8 μL sterile distilled PCR grade water was added to a final volume of 20 μL. The PCR program used was an initial denaturation (94 °C for 5 min), 45 cycles of denaturation (94 °C for 30 s), annealing (55 °C for 30 s), extension (72 °C for 1 min, 30 s) and final extension for (72 °C for 10 min).

### Analysis of the PCR products

The PCR product was electrophoresed on 1% agarose gel in 1 x Tris Borate Ethylenediaminetetraacetic acid (EDTA) buffer stained with gel red and was visualized under UV transilluminator to confirm that a fragment of the correct size had been amplified. Automated sequencing of 16S rRNA genes of the organism was done using the AB 35100 x L genetic analyzer (Thermo Fisher Scientific, USA). Sequencing reaction was performed according to the manufacturer’s protocol using Big Dye version 3.1 dye terminator cycle sequencing kit (Applied Biosystems) with 27f and 1492r primers. The sequence was aligned in the Gene bank database using Basic Local Alignment Search Tool (BLASTN) program at the National Centre for Biotechnology Information (NCBI) and percent homology score was obtained to identify the organism.

### Extraction and purification of bioflocculant

Adopting the modified methods of Chen et al. [18] and Piyo et al. [19], the purification of the extracted bioflocculant was performed. Briefly, after 72 h of fermentation, the culture broth was centrifuged (8000 rpm, 30 min) to remove bacteria cells. One volume of sterile distilled water was added to the supernatant and centrifuged at 8000 rpm for 15 min to remove insoluble substances. Two volumes of ethanol were later added to the supernatant, stirred and left to stand for 12 h at 4 °C. The precipitate obtained was vacuum-dried to obtain crude bioflocculant. The crude product was then dissolved in water to yield a solution, to which one volume of chloroform: n-butyl alcohol (5:2 v/v) was added. The mixture was stirred, poured into a separating funnel and allowed to stand for 12 h at room temperature. Finally, the supernatant was discarded and two volumes of ethanol were added to recover the precipitate and then lyophilized to obtain a purified bioflocculant.

### Jar test determination of bioflocculant dosage

Different concentrations (0.1 to 1.0 mg/mL) of the purified bioflocculant were prepared and their flocculating activities measured against 4 g/L kaolin clay suspension. Exactly 3.0 mL of 1% (w/v) CaCl_2_ was added to the different concentrations of the purified bioflocculant and mixed with 100 mL of kaolin clay suspension in 500 mL beakers. The solution was rapidly mixed at 160 rpm for 2 min, followed by gradual flocculation at 40 rpm for 2 min and sedimentation for 5 min. After sedimentation, 2 mL was gently withdrawn from the upper clarifying phase in order to measure the flocculating activity [20]. The concentration dosage that gave the best flocculating activity was used for subsequent experiment.

### Effect of cations on flocculating activity

The effect of different cations on bioflocculant production was assessed by replacing CaCl_2_ in the production medium with Na^+^, K^+^, Mg^2+^, Mn^2+^ Al^3+^ and Fe^3+^ using the method of Kurane et al. [17].

### Effect of pH on flocculating activity

The effect of pH on flocculating activity of bioflocculant produced was assessed by adjusting the pH of the production medium using 0.1 M HCl and 0.1 M NaOH at the pH range of 3-12. [10].

### Effect of temperature on flocculating activity

Heat stability was evaluated by incubating the bioflocculant solutions in water bath at a temperature range of 50, 60, 70, 80, 90 and 100 °C for 25 min. Afterwards, the residual flocculating activity was determined using the protocol of Gong et al. [21].

### Fourier transform infrared spectroscopy (FTIR) analysis

The purified bioflocculant was subjected to FTIR analysis using FTIR Spectrophotometer Attenuated Total Reflectance (FTIR-ATR) (Perkin Elmer Spectrum 100, USA). Exactly 5 mg of the dried bioflocculant was pulverized with potassium bromide and forced into bits for FTIR spectral measurement in the frequency range of 4000–650 cm^−1^.

### Thermo-Gravimetric Analysis (TGA) and SEM analysis

The pyrolysis profile of the purified bioflocculant was monitored with a Simultaneous Thermal Analyser (Perkin Elmer STA 6000 Germany, USA). The surface morphology structure of the purified bioflocculant was examined using Scanning Electron Microscopy (SEM) (JEOL-JSM-6390LV, Japan).

### Chemical composition of the bioflocculant

The total sugar content was determined by phenol-sulphuric acid method using glucose as standard [22]. The protein content of the purified bioflocculant was determined by the Lowry method with serum albumin (BSA) used as the standard [23].

### Lab-scale studies of bioflocculant produced by *Arthrobacter humicola* in sewage waste water treatment

The sewage waste water collected from the waste water treatment plant, Phuthaditjabha, Eastern Free State Province, South Africa, was used to validate the flocculating efficiency of the test bioflocculant. Different physiological parameters like pH, suspended solids, biological oxygen demand BOD, turbidity, COD and nitrate of the water sample were measured before and after treatment. Optimum dose of 0.8 mg/mL of the purified bioflocculant and 3 mL of 1% CaCl_2_ were added into 100 mL of sewage waste water. The contents were agitated at 160 rpm for 2 min using jar test and the speed was later reduced to 40 rpm for 2 min to facilitate floc formation. The treated samples were left to settle for 5 min and the supernatant was used for further physiological analyses. This procedure was conducted in triplicates. The turbidity, suspended solids, COD and nitrate were measured using spectrophotometer DR 3800 and turbidimiter (HACH, USA). The removal efficiencies were thereafter calculated as:

$$ R E=\left[\frac{A_o- A}{A_o}\right]\times 100 $$

Where Ao and A are the initial and final values obtained before and after treatment respectively.

To assay for the BOD, 25 mL of raw and 50 mL of treated waste water samples were added into BOD bottle and the bottles were filled up with BOD buffer (2.25% MgSO_4_.7H_2_O, 2.75% CaCl_2_, 0.025% FeCl_3_.6H_2_O and phosphate buffer solution). The BOD buffer was used as the initial working solution. The bottles were incubated at 20 °C for 5 days. The initial and final dissolved oxygen (DO) were measured after 15 min and 5 days respectively using a HI5421 BOD Meter (Hanna, USA). The BOD and the percentage BOD removal efficiency were subsequently estimated using the equations:

$$ BOD=\frac{D_1-{D}_2}{P} $$

D_1_ = DO in diluted specimen after preparation

D_2_ = DO after 5 days

P = decimal fraction of specimen used

$$ R E\left(\%\right)=\frac{B_1-{B}_2}{B_1}\times 100 $$

B_1_ = Untreated sample

B_2_ = Treated sample

### Statistical analysis

Results were expressed as mean value ± standard deviation of three replicates and were subjected to one way analysis of variance (ANOVA) followed by Duncan multiple range tests to determine significant differences in all the parameters using SPSS version 16.0. Values were considered statistically significant at *p* < 0.05.

## Results and discussion

### Molecular identification of bioflocculant producing organism

Ten actinobacteria strains were isolated from soil sediment of Sterkfontein dam and were assessed for flocculating efficiency. Strain SFD 07 which demonstrated highest flocculating efficiency of 85% against kaolin clay suspension was selected for further experiment. The 16S rDNA PCR yielded a product of expected size (approximately 1.5 kb). Basic Local Alignment Search Tool (BLAST) analysis of the nucleotide sequence of the 16S rDNA revealed the bacteria to have 99% similarity to *Arthrobacter humicola strain R1* and the sequence was deposited in the Gene bank as *Arthrobacter humicola* with accession number KC816574.1.

### Effect of bioflocculant dosage on flocculation

**Fig. 1:**
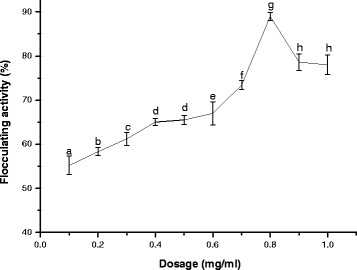
Effect of concentration on flocculating activity. Percentage flocculating activities with different alphabetic letters are significantly different (*p*<0.05)

The data obtained with respect to the flocculating efficiency over the dosage range of 0.1 to 1.0 mg/mL of the purified bioflocculant is shown in Fig. 1. The flocculating efficiency was a bit weaker at lower concentrations (0.1-0.7 mg/mL). It is however noteworthy that the maximum flocculating efficiency (89%) was observed at 0.8 mg/mL dose and this was choose as the best (optimal) concentration for the successive assays. Further increase in bioflocculant dosage resulted in a decline in flocculating activity. Insufficient dosage of bioflocculant will hinder or affect bridging mechanism formation of flocs and over dosage will result into high viscosity formation which will inhibit sedimentation of suspended particles by restabilising the kaolin particles. Hence, establishing the optimum bioflocculant dosage is an important parameter in flocculation. Our assertion in this study on the effect of dosage on flocculation is not only significant but also agrees with the report of Ugbenyen and Okoh [24], where the bioflocculant produced by the consortium of *Cobetia* spp and *Bacillus* sp attained its optimum flocculating activity of 90% at concentration dosage of 0.8 mg/mL. In contrast to the present study, the bioflocculant produced by *C. daeguense* was more than 90% in the range of 0.3-8.2 mg/L and the maximum value achieved 96.9% at bioflocculant dosage of 1.2 mg/L at optimal pH 5.6 and temperature of 15 °C [25].

### Effects of cations on bioflocculant production

The effect of metal ion on purified bioflocculant is shown in Table 1. Cation had played an important function in flocculation by neutralizing and stabilizing the negative charges of both functional groups of kaolin clay suspension and bioflocculant [26]. The results of this study demonstrated that all the cations tested stimulated the flocculating activity of the bioflocculant produced against kaolin clay suspension with the exception of monovalent cations. Interestingly, the highest flocculating activity was observed with divalent cations (Ca^2+^ and Mg^2+^) which agrees with the report of Kumar et al. [27] where flocculating efficiency of bioflocculant produced by a haloalkaliphic *Bacillus* sp. was enhanced in the presence of Ca^2+^, Cu^2+^ and Zn^2+^ which are representatives of divalent cations. It is noteworthy that the divalent cations appear to enhance the neutralization of negative charges on kaolin clay suspension and the bioflocculant thereby minimizing the gap in between and enhancing the adsorption of bioflocculant to the surface of the kaolin clay which led to agglomeration of flocs development and better sedimentation of the kaolin clay [28].

**Table 1:** Effect of cations on flocculating activity (*n*=3, mean ± SD)

CATIONS	FLOCCULATING ACTIVITY (%)
Fe^3+^	57.90 ± 0.4^a^
Al^3+^	65.30 ± 0.6^b^
K^+^	33.10 ± 1.6^c^
Mg^2+^	88.70 ± 0.9^d^
Na^+^	41.70 ± 1.5^e^
Mn^+^	75.70 ± 2.25^f^
Ca^2+^	89.00 ± 0.7^d^

Percentage flocculating activities with different superscripts are significantly different (*p*<0.05)

### Effect of pH

**Fig. 2:**
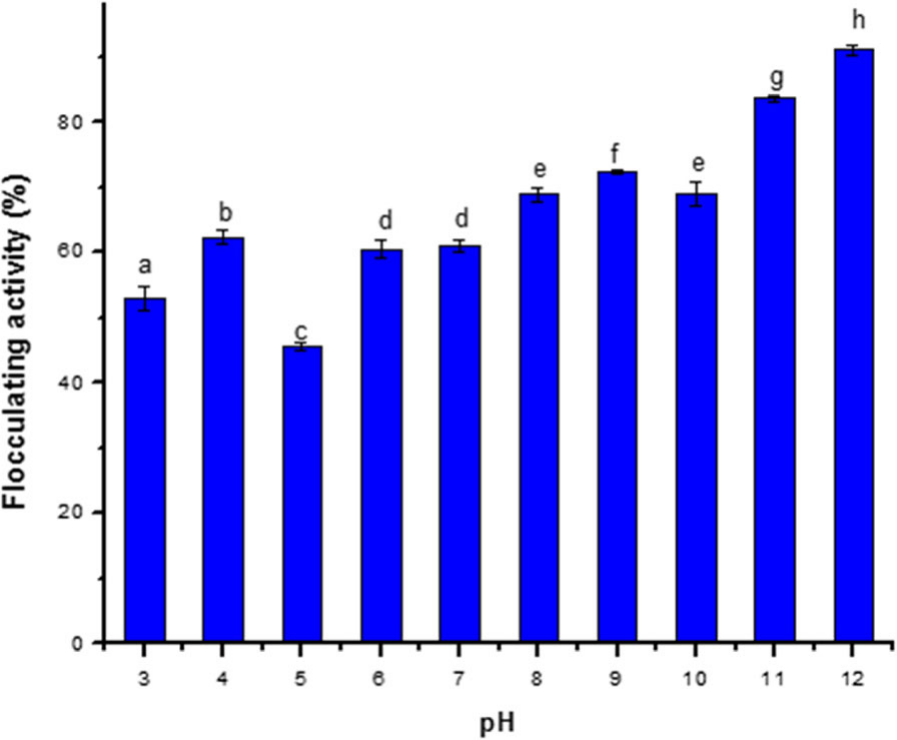
Effect of pH. Bars with different superscript for the parameter are significantly different (*p<0.05*)

The result of the influence of pH (over a range of 3-12 scale) on the flocculating activity is presented in Fig. 2. This study demonstrates that acidic and basic pH media support flocculating efficiency by the test organism, with the most pronounced activity observed in the basic medium. This observation corroborates the fact that bioflocculants exhibit varying degree of electrical states at different pH which impact on the flocculating activity of the bioflocculant for kaolin particles [29]. The best and most potent flocculating activity (91%) was observed at the highest pH scale value (pH 12) and this corroborates the report of Li et al. [30], where bioflocculant produced by *Arthrobacter* sp. B4 attained its maximum flocculating activity at pH 12. In another study, the alkaline pH range of 7-12 favoured the bioflocculant produced by *Bacillus megaterium* and maximum yield of bioflocculant was obtained at pH 9, while it was inhibited in an acidic culture medium [28].

### Effect of temperature

**Fig. 3:**
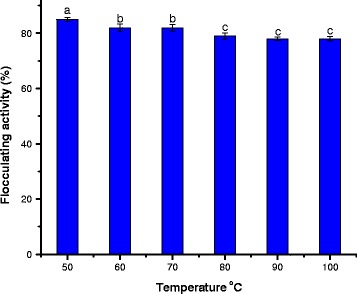
Thermal stability of bioflocculant produced by *Arthrobacter humicola*. Bars with different superscripts are significantly different (*p*<0.05)

The behaviour of the bioflocculant when exposed to heat is shown in Fig. 3. More than 80% flocculating activity was retained at 50-70 °C after heating the bioflocculant for 25 min. However, there was stability in the flocculating activity over a temperature range of 80-100 °C. It is noteworthy that based on the structure of bioflocculant, more than 78% flocculating activity was retained after heating the purified bioflocculant for 25 min at the highest temperature (100 °C). Hence, this result suggests that the main backbone of the bioflocculant is a polysaccharide. Likewise, the results of the biochemical analyses of the purified bioflocculant confirmed that 82% of polysaccharide were detected with no presence of protein in the structure. This observation is not only outstanding but also affirmed that the main backbone of the purified bioflocculant structure is a polysaccharide, which is supportive of its excellently elicited flocculating activities in this study. This is consistent with the reported findings that flocculants rich in polysaccharide are more heat stable than those composed of mainly protein and nucleic acids [10, 31, 32].

### Application of the purified bioflocculant in the treatment of sewage waste water

The discharge of untreated or partially treated sewage water containing non-biodegradable materials, heavy metals and other toxicants complicates the degree of contamination of the receiving water bodies. This challenge has necessitated the need for proper treatment of various effluents prior to discharge into receiving water body. In addition, sewage carries an array of pathogenic microbes of clinical importance. During the course of decomposing the organic materials in water bodies, the availability of dissolved oxygen gets depleted and can lead to the death of many species of fish and other aquatic life forms [33]. Having validated the flocculating efficiency of our test bioflocculant against kaolin clay suspension, its potential in treating sewage effluent waste water was examined and the physicochemical properties are presented in Table 2. From the results (Table 2), it is evident that the bioflocculant produced by *Arthrobacter humicola* significantly reduced the degree of turbidity and removed nitrate, COD, BOD and the total suspended solids at removal efficiencies of 81.3%, 71.4%, 65.7%, 63.5% and 55.7% respectively (Table 2). The use of microbial flocculants in the treatment of waste water has been well documented. For instance, the bioflocculant produced by *Bacillus mucilaginosus* was able to remarkably remove COD, BOD and suspended solids in domestic, brewery and pharmaceutical waste waters and was a suitable alternative to the synthetic flocculants [34]. Similarly, the bioflocculant produced from *A indicus* CAN was able to reduce BOD, COD and suspended in waste water samples in the range of 38-80%, 37-79% and 41-68% respectively at a bioflocculant dosage of 500 mg/L [35]. Hence, the observations on the purified bioflocculant used in this study are confirmative that the test bioflocculant could serve as an alternative coagulant to chemical flocculants and can be employed in waste water treatment prior to discharge into water bodies.

**Table 2:** Physicochemical characteristics of sewage waste water before and after treatment with purified bioflocculant

Parameter	BT	AT
pH	7.73 ± 0.24	7.88 ± 0.56
Turbidity (NTU)	128 ± 1.12	24.0 ± 0.84
SS (mgL^-1^)	201 ± 1.40	89.0 ± 1.23
Nitrate (mgL^-1^)	8.40 ± 0.27	2.40 ± 0.72
COD (mgL^-1^)	1360 ± 1.74	467 ± 1.32
BOD (mgL^-1^)	49.20 ± 0.18	17.92 ± 0.64

Values are expressed as means ± standard deviation of triplicate determinations

*NTU* Nephelometric turbidity units

*COD* Chemical oxygen demand

*BOD* Biological oxygen demand

*SS* Suspended solids

*BT* Before treatment

*AT* After treatment

### Fourier transform infrared spectroscopy of the purified bioflocculant

**Fig. 4:**
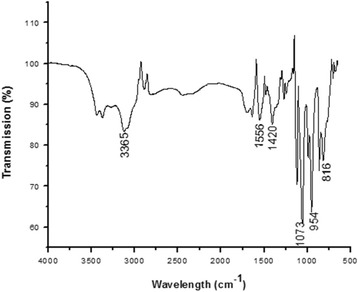
Fourier-transform infrared spectrogram of purified bioflocculant produced by *Arthrobacter humicola*

Presented in Fig. 4 is the result of the FTIR study of the investigated bioflocculant. The spectrum of the purified bioflocculant exhibited a band at 3365 cm^-1^ which is descriptive of a hydroxyl group. The band at 1420 cm^-1^ could be assigned to the symmetrical stretching in the carboxylate, indicating the presence of uronate in the purified bioflocculant [36]. The peak at 1073 cm^-1^ is characteristic of C-O groups suggesting the presence of carboxyl group in the purified bioflocculant [37]. Furthermore, the absorption bands at 954 and 816 cm^-1^ respectively are suggestive of sugar derivatives. Xiong et al. [10] reported that the small absorption peak is associated with B-glycosidic linkages and sugar monomers. The occurrence of carboxyl and hydroxyl functional groups suggests adsorption positions for suspended particles and has been opined to be the best choice of functional groups for flocculation process [38]. Interesting, the results of the biochemical analyses of the purified bioflocculant for carbohydrate and proteins revealed it contains 82% polysaccharide with no detection of protein in the structure. This observation is not only remarkable but also confirms that the main backbone of the purified bioflocculant structure is a polysaccharide, which is supportive of its excellently elicited flocculating activities in this study.

### Thermogravimetric analysis of purified bioflocculant

**Fig. 5:**
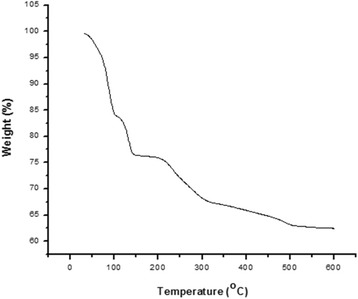
Thermogravimetric analysis of purified bioflocculant

The pyrolysis profile of the bioflocculant was examined using thermogravimetric analyzer. As shown in Fig. 5, there was a decomposition at 50 and 140 °C and about 77% weight was retained. The initial loss in weight could be attributed to moisture content of the bioflocculant. Furthermore, when the temperature was increased from 150 to 500 °C, there was a decrease of about 27% mass fraction which could occur as a release of volatile hydrocarbons from the heat decomposition of the polysaccharides in the purified bioflocculant [39]. However, further increase from 500 to 600 °C demonstrated that the purified bioflocculant weight was retained. Thus, affirming the main component of the material to be polysaccharide.

### SEM and EDX analysis

**Fig. 6:**
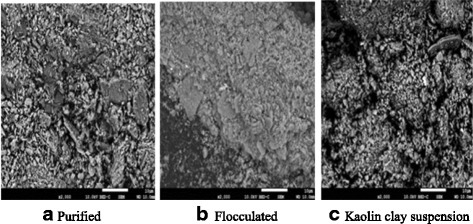
Scanning electron micrographs of **a** purified **b** flocculated **c** Kaolin clay suspension

The micrographs of the morphological structure of the purified bioflocculant and its interaction with kaolin clay suspension using Scanning Electron Microscope are shown in Fig. 6. While the purified bioflocculant appeared as whitish flakes structure (Fig. 6a), the interaction of the purified bioflocculant and the kaolin particles resulted into compacted floc formation (Fig. 6b). This was so because the kaolin particles were adsorbed on the binding sites of the purified bioflocculant and the subsequent interactions resulted into agglomeration of larger flocs (Fig. 6b). The EDX analysis confirmed the following proportions C: O: Na: P: K with 13.90: 41.96: 26.79: 16.61: 0.74 weight percentage respectively.

## Conclusions

The bioflocculant investigated in this study is made up of polysaccharide as its main backbone and demonstrates good flocculating efficiency against kaolin clay. FTIR spectral analysis demonstrated the presence of carboxyl and hydroxyl moieties as the major functional groups. The high flocculating activity demonstrated by the bioflocculant coupled with the removal of COD, BOD, turbidity, SS and nitrate at better efficiencies suggested its industrial application in waste water treatment. There are ongoing investigations within our research group on its oral toxicological assessment and large scale production.

## Abbreviations

BLAST: Basic local alignment search tool
BOD: Biological oxygen demand
BSA: Bovine serum albumin
COD: Chemical oxygen demand
DNA: Deoxyribonucleic acid
EDX: Energy dispersive X-ray
FA: Flocculating activity
FTIR: Fourier transformer infrared spectroscopy
NCBI: National center for biotechnological information
OD: Optical density
PCR: Polymerase chain reaction
RNA: Ribonucleic acid
SEM: Scanning electron microscope
TGA: Thermogravimetric analyzer
YMA: Yeast malt extract agar
